# Tolerability is more important than simplicity for treatment durability

**DOI:** 10.7448/IAS.17.4.19765

**Published:** 2014-11-02

**Authors:** Benoit Trottier, Nima Machouf, Emmanuelle Huchet, Stephane Lavoie, Sylvie Vezina, Michel Boissonnault, Louise Charest, Marie Munoz, Danielle Legault, Daniele Longpré, Réjean Thomas

**Affiliations:** 1 Clinical Research, Clinique Médicale l'Actuel, Montreal, Canada; 2 Epidemiology, Clinique Médicale l'Actuel, Montreal, Canada; 3 Interim Clinical, Clinique Médicale l'Actuel, Montreal, Canada; 4 Medicine, Clinique Médicale l'Actuel, Montreal, Canada; 5 Clinical, Clinique Médicale l'Actuel, Montreal, Canada; 6 Clinique Médicale l'Actuel, Montreal, Canada

## Abstract

**Introduction:**

Many studies have shown the superiority of single tablet regimens (STRs) of antiretrovirals for the treatment of HIV in terms of efficacy, adherence and rate of hospitalisation as they offer a low pill burden and once daily dosing. Our objective was to compare the duration of first-line STRs to multi-tablet regimens.

**Methods:**

From our clinical database, we selected patients initiating any of the major first-line regimens between 2007 and 2013. Two STRs, Atripla (ATP) and Complera (CPLR), were compared to three non-STRs: two NRTIs and raltegravir (RAL), atazanavir/ritonavir (ATV/r) or darunavir/ritonavir (DRV/r). The primary outcome was time to discontinuation of the first-line regimen. The association between regimen type and duration was estimated using Cox proportional hazards models adjusted for age, gender, baseline CD4, baseline viral load, risk factor, site and year of treatment initiation.

**Results:**

A total of 743 patients (281 on STRs and 462 on non-STRs) were included. 693 (93%) were male and median age was 43 years. Median length of follow-up was 3.2 years. 56% of patients were MSM, 6% IDU and 6% from endemic countries. Patients on an STR were less likely to be IDU (p<0.024) and have a baseline HIV-RNA ≥100,000 copies/mL (p<0.011). Overall, 321 (43%) patients discontinued their regimen during the study period. The rate of discontinuation one year after starting ARV depends on the regimen: 29% for patients on 2NRTIs+DRV/r, 26% on ATP, 25% on 2NRTIs+ATV/r, 17% on 2NRTIs+RAL and 10% on CPLR (p<0.001). In the adjusted model, durability for STR and non-STR was equivalent (aHR=0.83, p=0.108). Compared to patients on ATP, patients on CPLR were less likely to discontinue (HR=0.58, p=0.070). No difference between ATP and the other regimens was observed: HR for 2NRTIs+RAL=0.92 (p=0.66), 2NRTIs+ DRV/r=1.16 (p=0.36), 2NRTIs+ATV/r=1.11 (p=0.46).

**Conclusions:**

Our findings suggest that STRs do not necessarily result in a more durable treatment. Even with a higher pill burden and/or twice daily dosing, patients initiating therapy with RAL or boosted-PI based regimens were not more likely to discontinue the first-line regimen compared to patients on an STR. Among the STR subgroups, the regimen with better known tolerability conferred more durable treatment. Limitations included our inability to adjust for the patient's adherence to a given regimen.

